# Is Single‐Session Peroral Endoscopic Myotomy With Fundoplication Safe and Effective in Achalasia? A Systematic Review and Meta‐Analysis

**DOI:** 10.1002/deo2.70220

**Published:** 2025-10-08

**Authors:** Yusuf Kagzi, Abuzar Asif, Srinivas Reddy Puli

**Affiliations:** ^1^ University of Illinois College of Medicine Peoria Peoria Illinois USA

**Keywords:** achalasia, fundoplication, gastroesophageal reflux, peroral endoscopic myotomy, POEM‐F

## Abstract

**Background:**

Peroral endoscopic myotomy (POEM) is a minimally invasive treatment for achalasia but is often associated with gastroesophageal reflux disease (GERD). Adding fundoplication (POEM+F) may reduce reflux while maintaining the benefits of myotomy. This systematic review and meta‐analysis evaluate the efficacy and safety of POEM+F in achalasia patients.

**Methods:**

A comprehensive search of electronic databases and conference proceedings was conducted through November 2024 to identify studies on POEM+F. Pooled proportions were calculated using both fixed effects models. Heterogeneity was assessed via Cochran's Q test. Primary outcomes included technical success, Eckardt score improvement, and post‐procedure esophagitis. Secondary outcomes included procedure time, adverse events, wrap integrity, and hospital stay.

**Results:**

Nine studies with 202 patients were included. The pooled technical success rate was 94.80% (95% confidence interval [CI]: 91.37–97.40). Mean total procedure time was 110.51 min, with 52.04 min for fundoplication. Eckardt scores improved from 8.30 (95% CI: 6.83–9.76) pre‐operatively to 1.08 (95% CI: 0.33–2.50) post‐operatively. Post‐procedure, 11.52% (95% CI: 6.10–18.38) had abnormal acid exposure and 20.65% (95% CI: 14.47–27.61) developed esophagitis. An intact wrap was seen in 75.69% (95% CI: 58.55–89.40) on follow‐up endoscopy. Mean hospital stay was 2.27 days (95% CI: 1.09–3.45).

**Conclusions:**

POEM with fundoplication is a safe and effective treatment for achalasia, offering high technical success, symptom relief, and a low incidence of GERD.

## Graphical abstract

This meta‐analysis of nine studies (202 patients) demonstrates that peroral endoscopic myotomy with fundoplication (POEM‐F) achieves a technical success of 94.8% and significant symptom improvement (Eckardt score reduction from 8.3 to 1.1). Post‐procedural esophagitis occurred in 20.7% and wrap integrity was preserved in 75.7% on follow‐up, supporting POEM‐F as a safe and effective strategy to mitigate reflux while maintaining efficacy in achalasia.

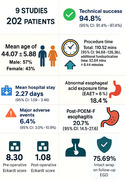

## 1 Introduction

Achalasia is a primary motor esophageal disorder characterized by the absence of relaxation of the lower esophageal sphincter (LES) and peristalsis along the esophageal body [1]. Patients often experience dysphagia, regurgitation, weight loss, chest pain, and heartburn. Three subtypes of achalasia include: type I (classic low intra‐esophageal pressure), type II (pan‐esophageal pressure), and type III (high‐amplitude premature spastic contractions). The treatment for achalasia depends on its subtype and includes options like pneumatic dilation, laparoscopic Heller myotomy (LHM), and peroral endoscopic myotomy (POEM). POEM has been a widely used, minimally invasive procedure for over 20 years [1, 2]. Although severe adverse events following POEM are rare, post‐op gastroesophageal reflux (GER) occurs in 8.5%–33% of cases. GER is a major long‐term side effect of POEM due to LES disruption, with esophagitis occurring in 13%–19% and abnormal acid exposure in 43%–47% of cases [3, 4, 5, 6]. It also increases the risk of Barrett's esophagus and esophageal adenocarcinoma, restricting the enthusiasm to treat achalasia with POEM [7].

Unlike LHM with fundoplication (LHM‐F), standard POEM does not include fundoplication, an anti‐reflux procedure to prevent post‐operative GER. In our study, post‐POEM GER was evaluated according to the Lyon Consensus criteria. Pathologic reflux was defined as an esophageal acid exposure time (EAET) greater than 6% on pH studies or endoscopic evidence of Grade B or higher esophagitis (i.e., Grade B, C, or D) [8]. Symptomatic GER and esophagitis after POEM are typically managed with proton pump inhibitors (PPIs), though clear guidelines for their use remain uncertain. Lifelong PPI therapy is recommended for Los Angeles (LA) grade C and D esophagitis, while 24‐h pH monitoring is advised for mild esophagitis and persistent symptoms [7]. Several modifications to the POEM technique have been attempted, such as avoiding posterior POEM, limiting gastric myotomy to <2.5 cm, and shortening the myotomy to <8 cm. However, none have significantly reduced post‐POEM reflux. In 2019, Inoue et al. introduced single‐session endoscopic partial fundoplication as a new extension of POEM [9]. POEM‐F involves dissecting the serosa, entering the peritoneal cavity, and creating a partial fundoplication by folding the gastric fundus and securing it to the distal myotomy. Over the past five years, this single‐session approach has gained attention, addressing both the functional and anatomical aspects of achalasia. It may help reduce symptoms, EAET, and PPI use in post‐POEM GER patients. However, its effectiveness and long‐term outcomes remain debated [7, 10].

This meta‐analysis evaluates the efficacy of single‐session POEM‐F for achalasia by assessing symptom relief, esophagitis reduction, safety, and technical success. The findings will help guide clinicians in optimizing treatment strategies.

## 2 Materials and Methods

### 2.1 Search Methodology

This systematic review and meta‐analysis were conducted and reported in accordance with the Preferred Reporting Items for Systematic Reviews and Meta‐Analyses (PRISMA) guidelines. From database inception to November 2024, a comprehensive literature search was performed using MEDLINE via PubMed, Ovid, Google Scholar, the Cochrane Library (Cochrane Central Register of Controlled Trials and Cochrane Database of Systematic Reviews), Embase, and the Database of Abstracts of Reviews of Effects (DARE). The following search terms were used: “POEM” OR “peroral endoscopic myotomy” AND “fundoplication” AND “achalasia.” Reference lists of included studies were reviewed for additional eligible articles. To minimize potential patient overlap, efforts were made to cross‐reference enrollment dates and study designs. While some studies originated from related institutions, no confirmed duplication of patient data was identified. In cases where original studies did not clearly define outcome thresholds, we applied widely accepted clinical definitions. For identifying and reporting significant post‐procedural major adverse events, the American Society for Gastrointestinal Endoscopy (ASGE) definitions were used as the standard [1]. For esophageal acid exposure, a threshold of >6% was used as per conventional criteria [7, 9]. These assumptions were necessary due to variability in reporting and are acknowledged as limitations.

### 2.2 Study Eligibility

Studies were included if they evaluated adult achalasia patients diagnosed via endoscopy and treated with single‐session POEM‐F. Non‐English articles, animal studies, case reports with fewer than three patients, editorials, and comments were excluded. Conference abstracts were eligible for inclusion if they reported on topics relevant to the review question. The database search was conducted by two authors (Yusuf Kagzi and Abuzar Asif), who independently reviewed the articles, and discrepancies were resolved through discussion.

Potential patient overlap, particularly among multicenter studies, was assessed by cross‐referencing enrollment dates, institutions, and study design.

### 2.3 Data Extraction and Quality Assessment

Two authors (Yusuf Kagzi and Abuzar Asif) independently extracted data into a standardized form, including (a) study details (primary author, study period, and publication year), (b) study design, (c) patient characteristics (sample size, demographics, follow‐up duration, and achalasia types), (d) intervention details (procedure time, additional fundoplication time), (e) outcomes (technical success, post‐op esophagitis risk, wrap integrity, and Eckardt score improvement), and (f) adverse events (erosion, gastric ulceration, and atelectasis).

### 2.4 Outcomes Evaluated

The primary outcomes included technical success (successful completion of POEM‐F), Eckardt score improvement, and the risk of post‐procedural reflux esophagitis. Secondary outcomes were total procedure time, additional fundoplication time, adverse events, wrap integrity, and abnormal EAET (defined as >6%).

### 2.5 Statistical Analysis

This meta‐analysis was performed by calculating weighted pooled effects. Individual study proportions were transformed into a quantity using the Freeman‐Tukey variant of the arcsine square‐root transformed proportion. The pooled proportion is calculated as the back‐transform of the weighted mean of the transformed proportions, using inverse arcsine variance weights for the fixed‐effects model and the random‐effects model [11, 12]. The heterogeneity of the studies was evaluated using Cochran's Q test, based on inverse variance weights, and by calculating the I^2^ statistic [13]. I^2^ values of 0–39% were considered as non‐significant heterogeneity, 30%–60% suggest moderate heterogeneity, 60%–75% substantial heterogeneity, and >75% considerable heterogeneity. A *p*‐value >0.10 rejects the null hypothesis that the studies are heterogeneous. The findings of this meta‐analysis are reported using the fixed‐effects model, as there was no statistically significant heterogeneity. Forest plots were drawn to show the point estimates in each study in relation to the summary of the pooled estimate. The width of the point estimates in the Forest plots indicates the weight assigned to that study. The Egger bias indicator and Begg‐Mazumdar bias indicator tested the effects of publication and selection bias on the summary estimates [14, 15]. Funnel plots were constructed to assess potential publication bias [16, 17]. For dichotomous outcomes, odds ratios (ORs) with 95% confidence intervals (CIs) were used. Microsoft Excel 19 was the software used to perform statistical calculations for this meta‐analysis.

## 3 Results

**FIGURE 1:**
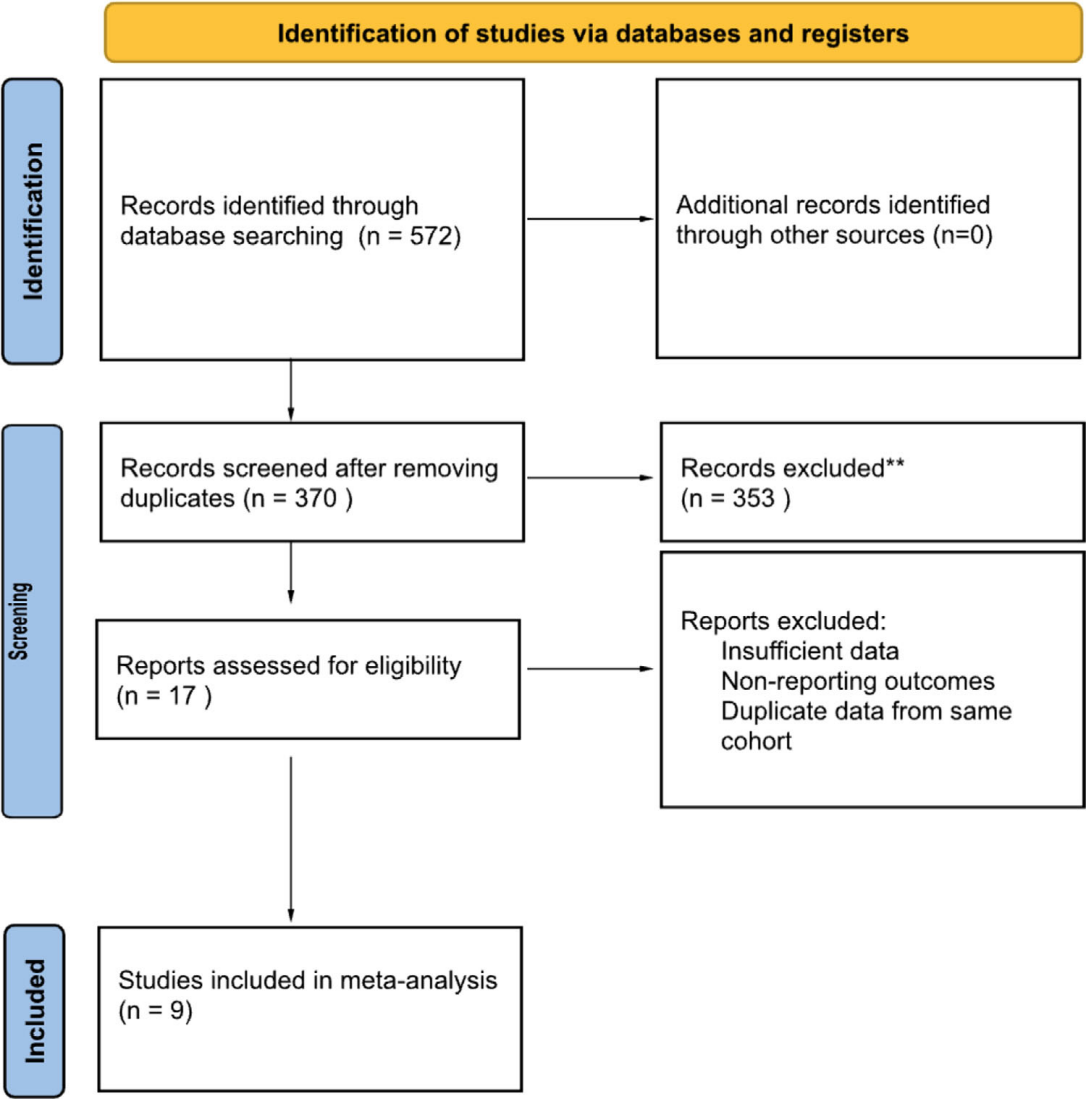
Preferred Reporting Items for Systematic Reviews and Meta‐Analyses (PRISMA) flow diagram depicting the selection process of studies included in the meta‐analysis.

An initial search identified 572 studies, of which 17 were deemed relevant for review. Data for this meta‐analysis were extracted from nine studies, pooling patient data from various sources that examined outcomes in achalasia cardia patients diagnosed through esophagogastroduodenoscopy (EGD) and high‐resolution manometry [8, 18, 19, 20, 21, 22, 23, 24, 25]. Data were extracted from four prospective and five retrospective cohorts, including three multicentric and six single‐centric studies. A PRISMA diagram with the details of the review process is shown in Figure 1. The quality of studies was evaluated using the Modified Newcastle‐Ottawa scale assessment tool for RCTs. As shown in Table 1, most studies were rated as good quality with scores ranging from 7 to 8 stars. All the pooled estimates given are estimates calculated using the fixed‐effects model. The estimates calculated using fixed‐ and random‐effects models were similar. The agreement between reviewers was 1.0, as measured by Cohen's κ.

**TABLE 1:** Modified Newcastle‐Ottawa Scale (NOS) Quality Assessment of the studies included in the meta‐analysis.

Study	Selection (max 4 stars)	Comparability (max 2 stars)	Outcome (max 3 stars)	Total Score (max 9 stars)	Quality Rating
Inoue et al., 2019	★★★★	★	★★★	8	Good
Bapaye et al., 2020	★★★★	★	★★	7	Good
Mandavdhare et al., 2020	★★	★	★	4	Fair (due to very small sample)
Patil et al., 2020	★★★	★	★★	6	Fair
Tyberg et al., 2022	★★★★	★	★★★	8	Good
Assefa et al., 2023	★★★★	★	★★	7	Good
Shrigiriwar et al., 2023	★★	★	★	4	Fair (small sample size)
Andalib et al., 2024	★★★	★	★★	6	Fair
Fayyaz et al., 2024	★★★	★	★★	6	Fair

### 3.1 Study Characteristics

A total of 202 patients underwent single‐session POEM‐F, with a mean age of 44.07 ± 5.88 years. Among them, 115 were male (57%) and 87 were female (43%). The mean duration of post‐POEM GER was 36 months. The mean follow‐up time post‐POEM‐F was 7 months, ranging from 1 to 21 months across studies.

### 3.2 Procedural Characteristics

The pooled mean total procedure time was 110.52 min (95% CI: 94.68–126.36). The pooled additional fundoplication time averaged 52.04 ± 6.44 min. The pooled mean hospital stay was 2.27 days (95% CI: 1.09–3.46).

### 3.3 Post POEM‐F Outcomes

#### 3.3.1 Technical Success

**FIGURE 2:**
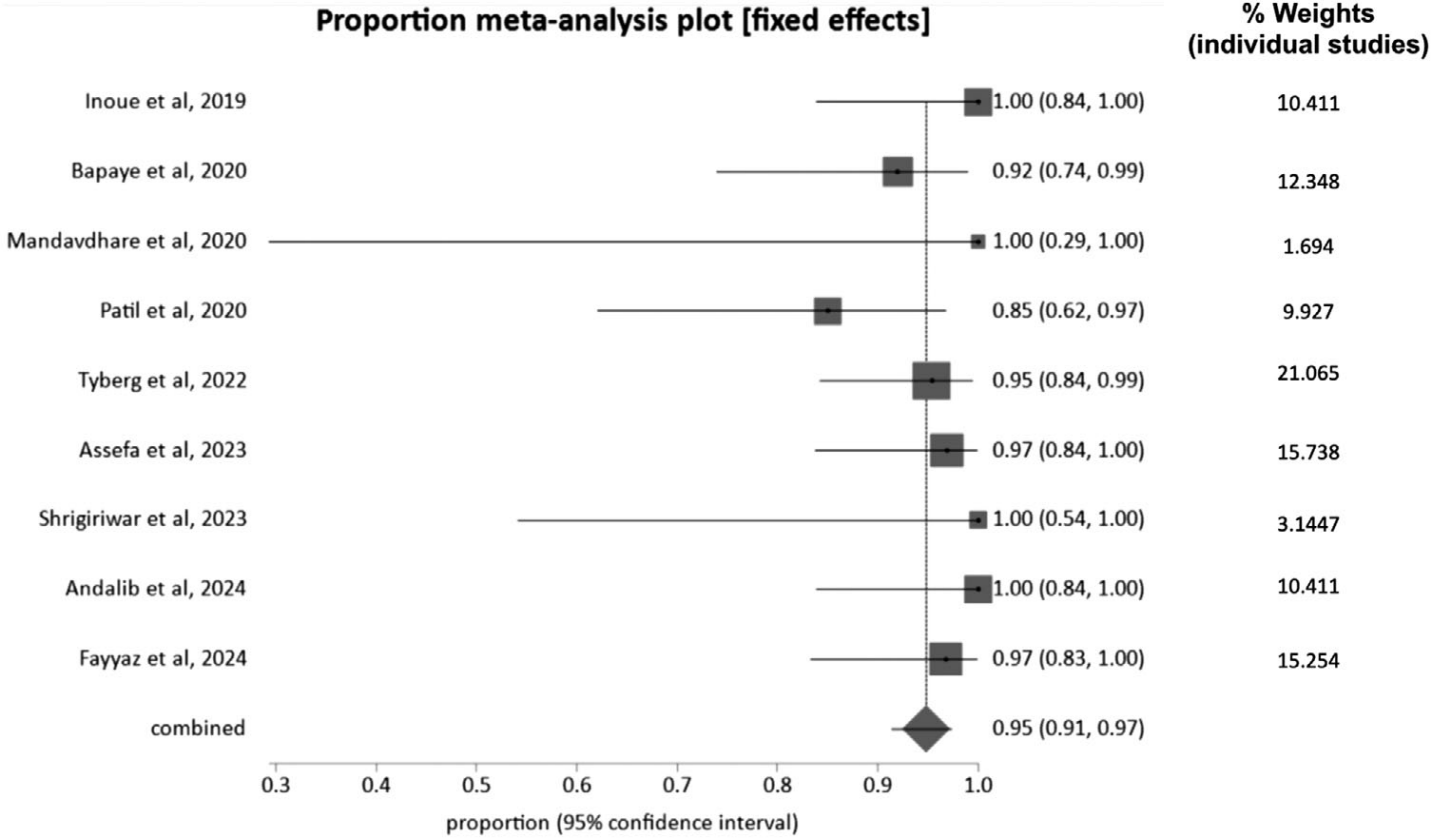
Forest plot showing individual study rates and the weighted pooled rate of technical success following peroral endoscopic myotomy with fundoplication (POEM‐F).

**FIGURE 3:**
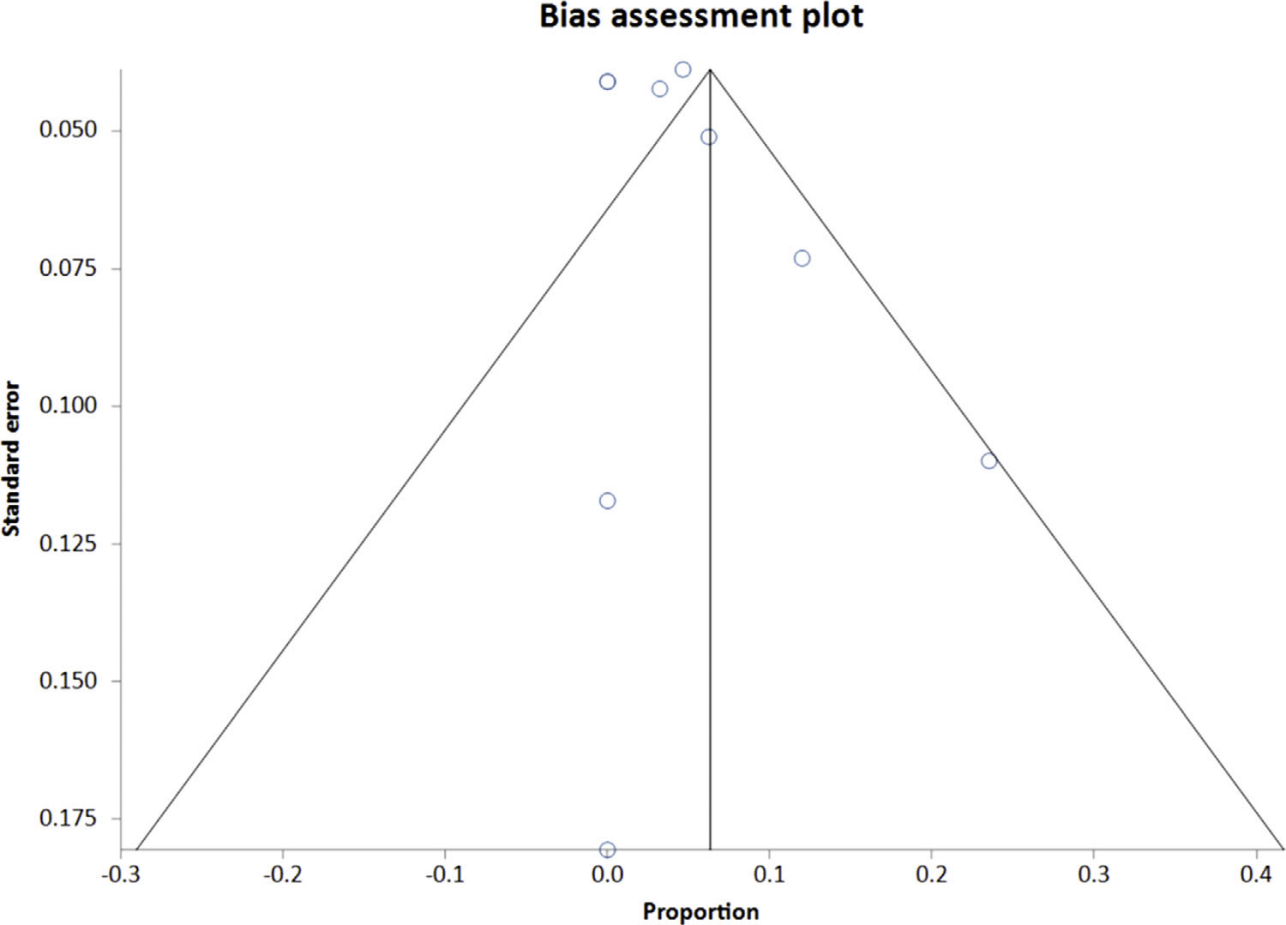
Funnel plot providing potential publication bias within the meta‐analysis using funnel plot asymmetry and regression‐based bias indicators.

Technical success was defined as the successful completion of POEM with fundoplication. Our analysis found a pooled success rate of 94.8% (95% CI: 91.4%–97.4%). There was no significant heterogeneity with an I^2^ = 0%. A forest plot displaying individual study estimates and the pooled estimate for overall technical success is shown in Figure 2. The Begg‐Mazumdar bias indicator yielded a Kendall's tau b value of 0.2 (*p* = 0.52), indicating no publication bias; however, given that fewer than 10 studies were included, this test has limited power, and the results should be interpreted with caution. A funnel plot assessing publication bias is shown in Figure 3.

#### 3.3.2 Improvement in Eckardt Score

The weighted mean Eckardt score decreased from a pre‐POEM‐F value of 8.30 (95% CI: 6.84–9.77) to 1.08 (95% CI: 0.33–2.50) after the procedure.

### 3.4 Post POEM‐F Follow‐up EGD Results

The pooled proportion of post‐POEM‐F esophagitis was 20.7% (95% CI: 14.5–27.6). Among studies reporting LA grade distributions, clinically significant esophagitis (grade B or higher) and severe GERD (grade C or higher) were documented. Patil et al. reported one case of grade B; Tyberg et al. reported one case of grade B and one case of grade C; Assefa et al. reported one case of grade C or higher; and Fayyaz et al. reported three cases of grade B, two cases of grade C, and one case of grade D. The pooled proportion of abnormal EAET (>6%) was 18.42% (95% CI: 3.25–42.83). Additionally, the pooled proportion of patients with intact wrap integrity on EGD after POEM‐F was 75.7% (95% CI: 58.6–89.4).

### 3.5 Post POEM‐F Adverse Events

**FIGURE 4:**
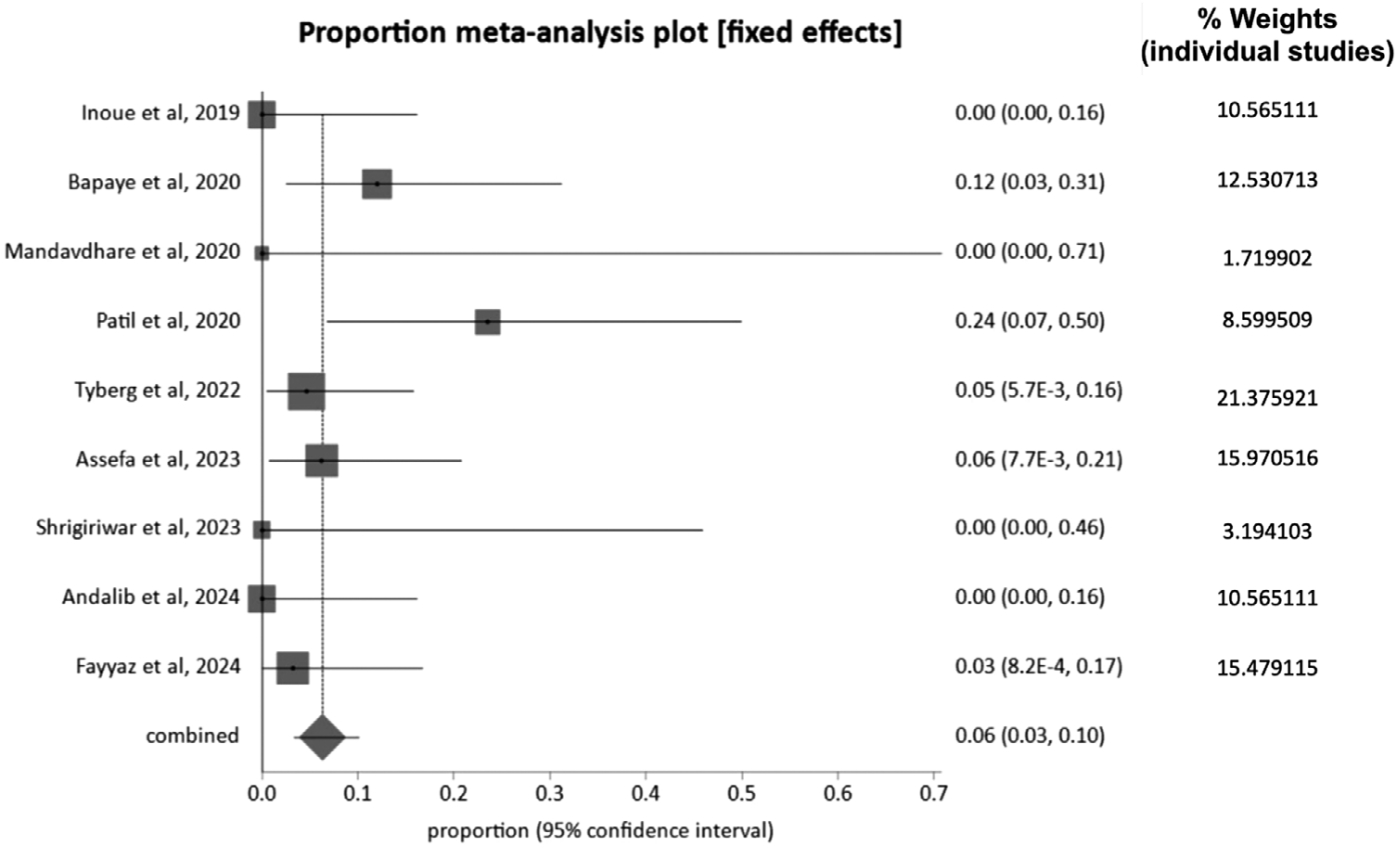
Forest plot showing individual study rates and the weighted pooled rate of overall adverse events following peroral endoscopic myotomy with fundoplication (POEM‐F).

Major adverse events, as defined by the American Society for Gastrointestinal Endoscopy, had a pooled proportion of 6.4% (95% CI: 3.0–10.9). Reported adverse events included endoclip and endoloop erosion, gastric ulceration, lung collapse requiring drainage, atelectasis, and mucosal injury. Expected events during the procedure, such as capno‐peritoneum and capno‐thorax, were excluded. A forest plot showing the individual study estimates and the pooled estimate of overall adverse events following POEM‐F is shown in Figure 4.

## 4 Discussion

Despite multiple studies since 2010 highlighting POEM as the preferred minimally invasive treatment for achalasia, managing post‐POEM GER has remained a challenge for both clinicians and patients in recent years [1, 26]. Since most patients are asymptomatic, the clinical significance of post‐POEM GER is often underestimated, raising concerns about potential undertreatment and the serious risks associated with pathological esophageal acid exposure [27]. A meta‐analysis found that 47% of patients who underwent POEM had abnormal ambulatory reflux exams, while only 8.5% reported GER symptoms [3]. Initially, the orientation of the myotomy in POEM was thought to influence GERD by preserving LES competency [26]. Medical management with PPIs appears helpful in controlling symptoms by altering the refluxate's composition, but does not impact its frequency or volume. Additionally, some patients experience refractory symptoms due to accelerated PPI metabolism caused by cytochrome P450‐2C19 polymorphisms with long‐term use. In this context, POEM‐F, introduced by Inoue et al. in 2019, has emerged as a promising alternative to the surgical LHM‐F to reduce post‐operative GER burden [8]. This meta‐analysis aims to evaluate the efficacy and safety of POEM‐F in patients with achalasia.

Our findings show that the technical success of POEM‐F was achieved in over 94% of patients. This success rate is similar to the >90% success rates reported for POEM alone and LHM with fundoplication (LHM+F) [28, 29]. Failures observed in the procedure were primarily due to technical issues and procedural side effects, rather than patient‐specific factors. Complications included difficulties with peritoneal cavity localization, device malfunctions, and severe esophageal dilation [18, 20, 25]. All patients who experienced failure with POEM‐F were successfully treated with POEM alone, without fundoplication.

Symptom severity in achalasia was measured using the Eckardt score, which assesses dysphagia, regurgitation, chest pain, and weight loss on a scale from 0 to 12. Clinical remission was defined as an Eckardt score of less than 3. Our analysis showed a significant reduction in the mean Eckardt score, from 8.3 to 1.08 in the POEM‐F group. A meta‐analysis of POEM also reported a similar trend, with the mean Eckardt score decreasing from 7 at baseline to below 2, further confirming the procedure's effectiveness in symptom relief [30].

EAET, a key objective measure of reflux, is the percentage of time the esophagus is exposed to stomach acid (pH < 4), as assessed through 24‐h ambulatory pH monitoring. An EAET greater than 6% of the total time confirms a diagnosis of GERD. An important finding in our study was that approximately 18% of patients had an abnormal EAET (>6%). This result is similar to the LHM‐F group (17%) but lower than that seen in the POEM‐alone group (39%) in previous studies [31]. A longer EAET is associated with more severe acid reflux symptoms and potential complications, such as esophagitis, strictures, Barrett's esophagus, and esophageal cancer. While EAET is an important objective measure of reflux, its clinical significance should be considered alongside symptomatic assessment and endoscopic findings [32].

Follow‐up endoscopy was used to assess reflux esophagitis based on the LA classification (per Lyon Consensus 2.0), ranging from grade A to D, which provides objective evidence of GERD requiring medical management [33, 34]. In our study, post‐POEM‐F esophagitis was observed in approximately 20% of patients. Repici et al. compared esophagitis rates between the POEM and LHM‐F groups, finding that the POEM group had an overall esophagitis rate of 35%, while the LHM‐F group had a lower rate of 7.6% [35]. Although the incidence of esophagitis after POEM‐F is lower than that seen with POEM alone, it remains higher than in the LHM‐F group, despite the addition of partial fundoplication, which aims to reduce the anatomical disruption caused by POEM. Several factors may explain this discrepancy. First, not all studies included in the analysis performed follow‐up endoscopy to assess esophagitis. Second, among studies that conducted second‐look endoscopy, the higher rate of esophagitis may be due to routine post‐POEM‐F evaluations, as it is a novel procedure that requires close monitoring. Third, given that our study had a mean follow‐up time of 7 months, early post‐operative endoscopic evaluations might show normal inflammatory changes that could be misinterpreted as esophagitis [35, 36]. Lastly, fewer than half of the patients in the previous LHM‐F studies underwent post‐operative endoscopy, which may account for the lower reported incidence of esophagitis. Recent guidelines suggest post‐procedural testing should be based on clinical suspicion rather than routine imaging, as increased surveillance may lead to false positives and weakly correlate with clinically significant outcomes [37, 38, 39].

Wrap integrity was another key parameter assessed in our analysis. Approximately 75% of patients showed an intact wrap on follow‐up EGD, which is comparable to the results reported in the LHM‐F group in previous studies [40]. Both techniques rely on surgical principles, such as tissue healing, fibrosis, and adhesion formation, to maintain wrap integrity and reduce the risk of reflux. In a few patients (*n* = 6), a loose wrap may have increased the likelihood of developing esophagitis. Further research is needed to assess the suturing techniques, such as endoscopic hand suturing, traditional endoloop with clips, and other suturing devices, may affect procedure time, technical success, and wrap durability. Ensuring a tighter and more durable wrap during endoscopic suturing could potentially enhance outcomes [18].

Although our analysis did not evaluate the impact of myotomy orientation or length on post‐POEM‐F reflux outcomes, prior studies provide some insights. Differences in approach, such as anterior versus posterior myotomy and the extent of gastric extension, have been reported with increased reflux risk, though their association with clinically significant esophagitis remains uncertain. A randomized trial comparing short (3 cm) versus long (≥6 cm) esophageal myotomies found higher rates of abnormal acid exposure (40%) and elevated DeMeester scores (56%) in the long myotomy group [40, 41]. Myotomy orientation, for instance, anterior myotomy has been reported to carry a lower risk of abnormal acid exposure compared with posterior myotomy involving incision of the angle of His (49% vs. 42%) [42].

In our study, major adverse events, as defined by the American Society for Gastrointestinal Endoscopy guidelines, occurred in 6% of patients who underwent POEM‐F. This rate was lower compared to the adverse event rates reported for POEM (13%) and LHM (25%) in previous studies. Bapaye et al. reported cases of erosions from endoclips (*n* = 1) and mucosal cuts from endoloops (*n* = 2), all of which healed fully without residual mucosal defects [17]. Similarly, Patil et al. documented a few cases of capnothorax (*n* = 4), with only one patient experiencing lung collapse that required a prophylactic intercostal drain for 24 h [20]. Other studies have observed nonspecific adverse events, such as chest pain, abdominal pain, transient fever, and superficial thrombophlebitis, all of which were managed effectively with conservative treatment and analgesics [9, 18, 19, 20, 21, 22, 23, 24, 25]. Importantly, no mortality was reported in any of the studies.

Single‐session POEM+F presents a promising alternative by combining the advantages of POEM with fundoplication in a single procedure, similar to the established surgical method of LHM+F. This approach is less reliant on devices, more cost‐effective, and aims to reduce post‐POEM GER, especially in asymptomatic cases. While technical challenges and occasional failures have been reported, these can be addressed through increased experience and continued refinement of techniques.

To our knowledge, Kamal et al. examined outcomes of POEM‐F, including four studies with 90 patients and focused primarily on feasibility and safety, reporting pooled technical success of 92% and esophagitis rates of 18%, with mean procedure and fundoplication times of 113 and 55 min, respectively [43]. More recently, Gopakumar et al. included seven studies with 127 patients, with an emphasis on mitigating post‐POEM GER, and reported technical success rates of 96.9% for POEM and 92.3% for fundoplication, wrap integrity of 84%, and composite clinical success of 86.2%; however, their review was not yet available at the time our search was conducted [44]. In contrast, our meta‐analysis incorporates a larger dataset of nine studies and 202 patients, providing an updated and more comprehensive evaluation. Beyond feasibility and safety, we evaluated additional clinically relevant outcomes, including improvement in Eckardt scores, post‐procedure acid exposure, and hospital stay.

However, there are a few limitations to this study. The number of studies included was small, and at least four of the included studies were available only as abstracts. The mean follow‐up duration across studies was 7 months, which limits assessment of long‐term effects such as GERD incidence, wrap integrity, and the durability of the fundoplication. As POEM‐F is a technically complex and evolving procedure, differences in institutional practice and operator experience may have influenced outcomes such as adverse events and wrap integrity. Although no definitive duplication of patient data was identified, a potential risk of overlap remains, particularly in multicenter studies from India and the United States, despite careful cross‐referencing of enrollment periods, participating centers, and study design. Although Kendall's tau analysis did not indicate evidence of publication bias, this finding should be interpreted cautiously, as statistical tests for publication bias are underpowered when fewer than 10 studies are available in a meta‐analysis. Further large‐scale, multi‐center randomized controlled trials are necessary to validate these findings and investigate additional factors that may affect treatment efficacy, such as achalasia subtype, patient age, and disease duration. As one of the aims of POEM‐F is to minimize the need for long‐term acid‐suppressive therapy, reporting of PPI use with longer follow‐up is needed to better assess the effectiveness of this approach. A subgroup analysis by achalasia subtype was not performed due to limited data, as type III achalasia patients often require longer myotomy, potentially affecting procedure time and GER risk. Additionally, exploring the long‐term effects on esophageal function and acid reflux is essential to optimizing patient care and minimizing complications related to achalasia management. While the risks are low, offering POEM‐F to all patients may be excessive due to the procedure's technical complexity and associated risks [45]. The greatest value of POEM‐F may lie in subgroups such as younger patients with an increased risk of reflux after standard POEM, those with a baseline hiatal hernia or other anatomic factors predisposing to GERD, or those with reflux not adequately controlled by PPIs may particularly benefit from the addition of fundoplication. Although current evidence is insufficient to define selection criteria, future studies should focus on identifying patient profiles most likely to benefit from POEM‐F. Despite these limitations, our meta‐analysis provides valuable insights into the safety of POEM‐F based on the current available evidence.

## 5 Conclusions

In conclusion, while no consensus currently exists on the optimal approach to prevent post‐POEM GER, single‐session POEM with fundoplication is a safe and effective treatment for achalasia. It offers high success, symptom relief, and a low incidence of post‐procedure GERD.

## Author Contributions

**Yusuf Kagzi**: Conceptualisation, Data curation, Methodology, Writing original draft; **Abuzar Asif**: projectAdministration, supervision, writingOriginalDraft; **Srinivas Reddy Puli**: Supervision, critical revision for intellectual content, and final approval of the version to be published.

## Conflicts of Interest

The authors declare no conflicts of interest.

## Ethics Statement

This study is a meta‐analysis of previously published data and does not involve direct patient participation or new human subject research. Therefore, ethical approval and informed consent were not required.
